# A Two‐Hit Hypothesis for Chemotherapy‐Induced Primary Ovarian Insufficiency in Asian Populations: A Population‐Specific Mechanistic Framework Linking Genetic Susceptibility and Cytotoxic Stress via the PI3K‐AKT‐FOXO3 Axis

**DOI:** 10.1155/bmri/2340719

**Published:** 2026-07-25

**Authors:** Zahra Dashti

**Affiliations:** ^1^ Faculty of Nursing and Midwifery, Islamic Azad University, Bojnourd Branch, Bojnourd, Iran, azad.ac.ir

**Keywords:** asian populations, autophagy, chemotherapy-induced ovarian toxicity, FOXO3, longevity, ovarian aging, oxidative stress, PI3K/AKT signaling, primary ovarian insufficiency

## Abstract

As cancer incidence rises in Asian countries, chemotherapy remains central to treatment amid rapid population aging and declining fertility in most of them. These trends underscore concern over long‐term reproductive health, as chemotherapy‐induced ovarian toxicity and premature ovarian insufficiency (POI) emerge as major late effects with unclear population‐specific susceptibility. This review examines chemotherapy‐induced ovarian damage via the PI3K‐AKT‐FOXO3 signaling axis, a key regulator of follicular quiescence, stress responses, and ovarian longevity. Evidence was synthesized from human studies, experimental models, and mechanistic investigations to develop this population‐specific conceptual framework. Chemotherapy initiates DNA damage and oxidative stress, which subsequently activate interconnected pathways involving mitochondrial dysfunction, dysregulated autophagy, apoptosis, ferroptosis, inflammatory signaling, and dysregulation of the PI3K‐AKT‐FOXO3 axis, ultimately accelerating follicular activation and depletion. A distinctive contribution of this review is the integration of longevity‐associated genetic susceptibility with ovarian vulnerability, highlighting evidence suggesting that certain FOXO3 and PI3K‐AKT pathway variants, reported to be more prevalent in several Asian populations, may influence susceptibility to chemotherapy‐induced ovarian injury. This review proposes a two‐hit hypothesis as a conceptual framework integrating currently available molecular, experimental, and population‐based evidence while acknowledging that direct clinical validation remains limited. Within this framework, longevity‐associated genetic predisposition affecting the PI3K‐AKT‐FOXO3 axis constitutes the first hit, whereas chemotherapy‐induced cellular stress represents the second hit, together accelerating follicular burnout and increasing the risk of POI. This framework supports future evaluation of genotype‐informed risk stratification, individualized fertility preservation strategies, and prospective validation in Asian cancer cohorts, with the ultimate goal of informing ethnicity‐specific fertility preservation strategies and optimized chemotherapy protocols.

## 1. Introduction

Cancer remains one of the leading causes of morbidity and mortality worldwide, with its burden continuing to rise despite substantial advances in early detection and treatment [1]. In 2022, approximately 19.96 million new cancer cases were diagnosed globally, leading to nearly 9 million deaths [2]. The increasing number of cancer survivors has shifted attention beyond survival toward long‐term treatment‐related complications, including preservation of reproductive health and quality of life [3–5]. Before the age of 70, cancer ranked as the first or second leading cause of death in 112 of 183 countries, and as the third or fourth leading cause in an additional 23 countries [6].

Although cancer incidence rates (defined as new cases per population) are generally two to three times higher in developed nations compared with developing ones, the sheer size of populations in Asian countries, such as India, results in a substantial overall burden. In India, for example, cancer cases are projected to rise from 1.32 million in 2020 to 2.08 million by 2040, representing a 57.5% increase [7]. By contrast, cancer‐related mortality in the United States fell by roughly 29% between 1991 and 2017, largely thanks to advances in early detection and more effective treatments [8]. In Korea, cancer has remained the leading cause of death since 1983 [9]. These statistics underscore that, despite regional variations, the rising number of cancer survivors worldwide highlights the urgent need to address the long‐term consequences of cancer treatment [7, 9–11]. Among these, reproductive dysfunction has emerged as a particularly pressing concern, especially for premenopausal women undergoing chemotherapy [12].

Chemotherapy, a cornerstone of cancer management, is well recognized for its gonadotoxic effects and is a leading cause of premature ovarian failure (POF). It affects approximately one‐third of women diagnosed with cancer before the age of 40, who experience treatment‐related ovarian insufficiency [8, 13, 14]. Numerous studies have demonstrated that chemotherapy adversely affects ovarian function in an age‐dependent and dose‐dependent manner, resulting in diminished ovarian reserve, impaired fertility, and structural damage to ovarian tissue [8, 15–17]. Current evidence indicates that chemotherapy‐induced ovarian injury is not mediated by a single mechanism but results from a coordinated network involving DNA damage responses, oxidative stress, mitochondrial dysfunction, inflammatory signaling, and multiple regulated cell‐death pathways [18–23]. Beyond reproductive impairment, female cancer survivors face higher risks of accelerated bone loss, sexual dysfunction, and cardiovascular morbidity compared with age‐matched women without a cancer history [24–26]. Further underscoring the systemic impact of chemotherapy‐induced ovarian damage [27].

As the central organ of female reproduction, the ovary plays a critical role in oocyte maturation and endocrine regulation. Premature loss of ovarian function not only compromises fertility but also precipitates early‐onset menopausal symptoms, significantly reducing long‐term quality of life. Consequently, preservation of ovarian function has become an integral component of survivorship care in young female patients undergoing chemotherapy. Current evidence indicates that approximately 70%–80% of women experience some degree of ovarian dysfunction following completion of oncological treatment, with many progressing to irreversible reproductive failure.

The loss of fertility can have profound psychological consequences, particularly for women who wish to conceive biological children [28]. Identifying populations at heightened risk for chemotherapy‐induced POF is therefore essential for improving fertility preservation strategies and optimizing survivorship care. This issue is particularly relevant in Asia, where the absolute burden of cancer continues to increase because of population growth, demographic aging, and improved survival, resulting in a growing number of reproductive‐age cancer survivors [4, 7, 11, 29–33]. At the same time, population aging and persistently declining fertility rates present major demographic challenges across the region. Although cancer remains a leading cause of premature mortality, its impact extends beyond survival to long‐term health outcomes, including reproductive function among survivors [6, 27, 34, 35]. Emerging evidence further suggests that susceptibility to chemotherapy‐induced toxicity varies among individuals and populations, reflecting complex interactions between genetic background, environmental influences, and treatment‐related factors [36, 37].

Population‐based genetic studies on longevity and healthy aging have identified several variants within the Phosphoinositide 3‐Kinase/protein kinase B/Forkhead box O3 (PI3K‐AKT‐FOXO3) signaling pathway that are reported to be more prevalent in certain Asian populations, suggesting potential population‐specific differences in regulation of cellular stress responses and tissue homeostasis. This pathway plays a fundamental role in maintaining ovarian follicle dormancy, regulating cell survival, coordinating DNA damage responses, and orchestrating cellular adaptation to stress. Collectively, these observations raise the possibility that inherited variation affecting the PI3K‐AKT‐FOXO3 signaling axis may contribute to interindividual differences in ovarian vulnerability following chemotherapy, particularly within certain Asian populations [38, 39]. However, the population‐specific contribution of this signaling pathway to chemotherapy‐induced ovarian damage and subsequent fertility outcomes remains poorly defined.

This review synthesizes current molecular, experimental, and population‐based evidence regarding the PI3K‐AKT‐FOXO3 signaling axis and its role in chemotherapy‐induced ovarian toxicity, with particular emphasis on Asian populations. Building upon this evidence, we propose a population‐specific two‐hit hypothesis in which inherited susceptibility affecting the PI3K‐AKT‐FOXO3 axis constitutes the first hit, whereas chemotherapy‐induced molecular stress acts as the second hit, together increasing the likelihood of follicular depletion and premature ovarian insufficiency (POI). Rather than implying definitive causality, this framework is intended to integrate existing evidence, provide a biologically plausible explanation for interindividual variability, and identify priorities for future mechanistic and prospective clinical studies.

## 2. Methodology

This study was conducted as a structured narrative review integrating evidence from human clinical studies, animal models, and mechanistic investigations related to ovarian physiology, chemotherapy‐induced ovarian toxicity, and POI.

A comprehensive literature search identified 230 potentially relevant records published from 2004 onward, retrieved from PubMed, Web of Science, and Google Scholar. To ensure clinical relevance and reflect contemporary advances in molecular and translational research, the final synthesis placed particular emphasis on studies published from 2015 onward, whereas key landmark studies published before 2015 were retained when they provided foundational evidence for ovarian physiology or PI3K/AKT/FOXO3 signaling.

The search strategy employed predefined keywords and, where appropriate, Medical Subject Headings (MeSH). Core terms included FOXO3, AKT, PI3K, chemotherapy, ovary, POI, oxidative stress, aging/longevity, cancer, and Asian populations. Boolean operators were applied to refine and combine concepts. Representative search queries included are as follows:

• (“oxidative stress” OR reactive oxygen species [ROS]) AND (ovary OR ovarian OR GC)
• (chemotherapy OR anticancer agents OR gonadotoxic therapy) AND (“primary ovarian insufficiency” OR POI OR POF OR “chemotherapy‐induced amenorrhea”)
• (chemotherapy) AND (“ovarian reserve” OR follicle depletion OR folliculogenesis) AND (Asian populations OR East Asian OR South Asian)
• (longevity OR aging OR lifespan) AND (FOXO3 OR FOXO3A OR PI3K/AKT/FOXO3 axis) AND (Asian populations)
• (chemotherapy‐induced ovarian toxicity) AND (population differences OR ethnic differences)

Studies were screened independently according to predefined eligibility criteria. Duplicate records, conference abstracts without full text, editorials, commentaries, studies unrelated to ovarian biology or chemotherapy‐induced ovarian injury, and articles lacking sufficient mechanistic or clinical relevance were excluded. Only peer‐reviewed publications published in English were considered eligible.

Eligible studies encompassed:

• human clinical and population‐based studies evaluating ovarian reserve, fertility outcomes, or genetic variants associated with chemotherapy exposure or reproductive aging.
• animal and experimental models examining chemotherapy‐induced ovarian injury or protective mechanisms.
• mechanistic studies investigating dysfunctional and protective signaling pathways in POI, including autophagy, oxidative stress, apoptosis, ferroptosis, DNA damage responses, inflammatory signaling, and FOXO3/AKT signaling.

Data extracted from eligible studies included study design, experimental model or study population, principal molecular pathways investigated, chemotherapy agents evaluated, major reproductive outcomes, and the principal mechanistic findings relevant to ovarian toxicity.

The majority of included studies focused on Asian populations, with particular representation from East and South Asia, including cohorts from China, Japan, South Korea, India, and Southeast Asian countries. Owing to the limited availability of high‐quality epidemiological and genetic data from low‐ and middle‐income countries, studies from these regions were comparatively underrepresented.

To place population‐specific findings within a broader context, comparative evidence from non‐Asian populations, including selected European and African cohorts, was also reviewed. However, because this review was designed as a structured narrative synthesis rather than a formal systematic review or meta‐analysis, no quantitative pooling of results or statistical assessment of publication bias was performed. Instead, emphasis was placed on consistency of evidence across experimental, clinical, and population‐based studies. For the same reason, formal risk‐of‐bias tools developed for interventional studies (e.g., Cochrane RoB2, ROBINS‐I) were not applicable to this review. Instead, the overall quality and transparency of the review process were self‐assessed using the scale for the assessment of narrative review articles (SANRA), a validated six‐item instrument specifically developed for narrative reviews, covering justification of importance, statement of aims, literature‐search description, referencing, scientific reasoning, and appropriate presentation of evidence [40].

**Figure 1:**
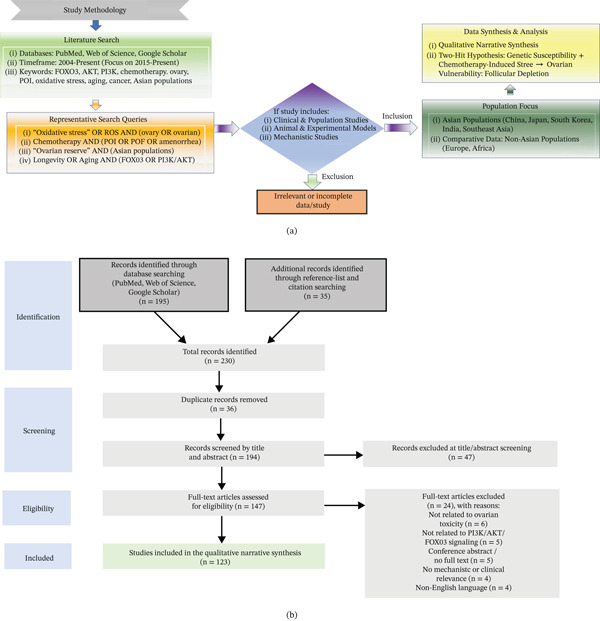
Overview of the review methodology. (a) Flow chart of study methodology for this review. Created by the author. (b) PRISMA‐style flow diagram illustrating the literature search and study selection process used in this review.

The methodological workflow was adapted from the PRISMA 2020 reporting framework to improve transparency in study identification, screening, eligibility assessment, and evidence synthesis. The study selection process is summarized in Figure 1b. In addition, the completed PRISMA 2020 checklist is provided in Table S1—PRISMA.

The overall methodological framework used to organize the review is illustrated in Figure 1a. The extracted evidence was subsequently synthesized to support the proposed two‐hit hypothesis, integrating genetic and longevity‐associated susceptibility with chemotherapy‐induced cellular stress into a unified mechanistic framework to explain heightened ovarian vulnerability and accelerated follicular depletion in Asian populations.

## 3. Discussion

This section reviews the molecular mechanisms that maintain ovarian homeostasis and regulate follicular development, followed by an integrated discussion of how chemotherapy disrupts these regulatory networks to induce ovarian injury. Among these regulatory pathways, the PI3K‐AKT‐FOXO3 signaling axis plays a central role in maintaining primordial follicle dormancy, preserving granulosa cell (GC) survival, and protecting the ovarian reserve from premature depletion. Dysregulation of this pathway promotes inappropriate follicular activation and accelerates loss of reproductive potential. The following sections examine the interconnected molecular mechanisms underlying chemotherapy‐induced ovarian toxicity, including DNA damage responses, oxidative stress, apoptosis, ferroptosis, autophagy, and dysregulation of intracellular signaling pathways. Finally, the potential clinical implications of these mechanisms are discussed in the context of POI, fertility preservation, and the proposed population‐specific two‐hit hypothesis in Asian women.

### 3.1. Ovarian Function and Follicular Regulation

As the primary female reproductive gland, the ovary plays a fundamental role in the production of mature oocytes and the secretion of sex hormones, thereby coordinating follicular growth and maturation across the reproductive lifespan [41, 42]. Primordial follicles constitute a finite and nonrenewable ovarian reserve and exhibit marked sensitivity to environmental insults and chemotherapeutic exposure [43, 44].

This vulnerability is most pronounced during the earliest stages of follicular development. It coincides with meiotic entry of oocytes and the onset of GC proliferation. Accordingly, the transition of follicles from a quiescent state to primary and preantral stages represents a critical window during which reproductive capacity is particularly susceptible to environmental and treatment‐related stressors. Maintenance of this quiescent state requires coordinated regulation of DNA integrity, cellular metabolism, oxidative balance, and survival signaling, emphasizing that follicular homeostasis depends on the interaction of multiple molecular pathways rather than a single regulatory mechanism [45].

Clinically, ovarian reserve is assessed using an integrated combination of demographic, sonographic, and endocrine parameters. These include maternal age, antral follicle count (AFC) measured by transvaginal ultrasound, circulating levels of anti‐Mullerian hormone (AMH), estradiol (E2), and follicle‐stimulating hormone (FSH), together with the derived FSH/LH (Luteinizing Hormone) ratio [41, 46, 47].

At the molecular level, FOXO3a emerges as a main regulator of ovarian homeostasis. By restraining excessive primordial follicle activation and limiting apoptosis in GCs, FOXO3a preserves follicular integrity. These effects are mediated, in part, through negative regulation of AKT and its downstream signaling components, with the endogenous AKT‐FOXO3a complex serving as a key physiological checkpoint controlling follicular atresia and GC survival [48]. Beyond regulating follicular dormancy, FOXO3 also functions as a key transcriptional mediator of cellular stress responses by integrating signals derived from oxidative stress, nutrient availability, and DNA damage [49]. This axis is further modulated by E2, which activates AKT signaling and promotes FOXO3a phosphorylation. As a consequence, FOXO3a is excluded from the nucleus, resulting in attenuation of its proapoptotic transcriptional activity in GCs [50].

More broadly, FOXO proteins function as principal downstream effectors of the PI3K/AKT pathway, translating phosphorylation‐dependent cues into transcriptional programs that govern proliferation, apoptosis, and cell‐cycle arrest [51, 52]. Recent studies further indicate that FOXO3 activity is dynamically regulated in response to genotoxic stress, allowing damaged oocytes either to initiate DNA repair programs, when damage exceeds repair capacity, or to undergo programmed cell death. Sustained FOXO3a activity is associated with delayed follicular and oocyte growth, as well as reduced oocyte volume.

In contrast, activation of the PI3K pathway drives FOXO3a phosphorylation and cytoplasmic translocation, leading to loss of its inhibitory function. This shift initiates follicular activation and supports oocyte growth, primordial follicle development, and GC proliferation. Chemotherapeutic agents disrupt this tightly regulated balance by simultaneously inducing DNA double‐strand breaks, excessive reactive oxygen species production, mitochondrial dysfunction, and inflammatory signaling, thereby altering PI3K‐AKT‐FOXO3 activity and compromising follicular survival. Mechanistically, AKT‐mediated phosphorylation suppresses FOXO3 nuclear localization and inhibits proapoptotic gene transcription [34, 53, 54]. Consequently, FOXO3 no longer functions solely as a regulator of follicular dormancy but also becomes an important mediator linking DNA damage responses with apoptotic signaling. Conversely, dephosphorylated FOXO3 translocates to the nucleus and activates apoptotic pathways characterized by mitochondrial membrane depolarization, cytochrome c release, caspase activation, and downregulation of the antiapoptotic protein B‐cell lymphoma 2 (Bcl‐2) [34, 55, 56].

By coordinating oocyte recruitment from the primordial follicle pool and maintaining granulosa and theca cell homeostasis, the PTEN/PI3K/AKT/FOXO3a axis plays a decisive role in safeguarding ovarian reserve. Within this tightly controlled framework, FOXO3a acts as a molecular gatekeeper, finely balancing follicular quiescence and activation and thereby protecting reproductive potential from premature depletion [57]. Disruption of this regulatory checkpoint is increasingly recognized as one of the earliest molecular events contributing to chemotherapy‐induced POI.

Under physiological conditions, survival and death signaling pathways remain tightly balanced to preserve follicular homeostasis. Chemotherapy disrupts this equilibrium by simultaneously suppressing protective pathways, including AMPK‐SIRT1 signaling, while activating stress‐responsive mediators such as JNK. The resulting shift toward oxidative stress, mitochondrial dysfunction, DNA damage signaling, and apoptosis establishes the molecular basis for accelerated follicular depletion [58–61].

### 3.2. Impact of Chemotherapy on Ovarian Function and Mechanisms of Injury

**Figure 2:**
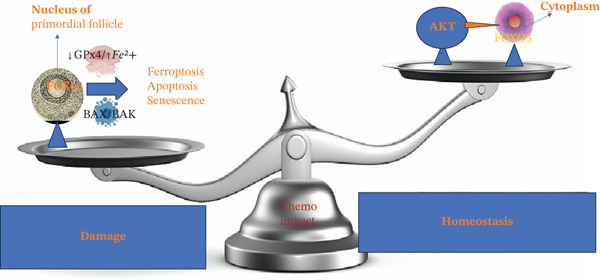
Illustration of how chemotherapy perturbs ovarian homeostasis by dysregulating the PI3K‐AKT‐FOXO3 signaling axis. Created by the author based on evidence synthesized from [8, 18–21, 23, 26, 27, 34, 36, 52, 62–66].

As a cornerstone of modern oncological treatment, chemotherapy effectively suppresses tumor cell proliferation and has markedly improved patient survival. Chemotherapy‐induced ovarian injury arises from multiple interconnected molecular mechanisms rather than a single pathogenic pathway. These mechanisms converge to disrupt follicular homeostasis and progressively deplete the ovarian reserve. As summarized in Figure 2, these interconnected events include DNA damage responses, oxidative stress, mitochondrial dysfunction, apoptosis, ferroptosis, inflammatory signaling, and dysregulation of the PI3K‐AKT‐FOXO3 pathway [62, 63]. Despite these benefits, its limited selectivity inevitably results in collateral damage to healthy tissues. Among the most vulnerable organs are the ovaries, where chemotherapy‐induced injury can profoundly compromise reproductive potential [8, 26, 64]. This heightened sensitivity reflects the finite nature of the ovarian follicular reserve, together with the exceptional vulnerability of oocytes and GCs to cytotoxic stress [27].

#### 3.2.1. Types of Chemotherapy Drugs and their Cellular Targets

Chemotherapeutic agents are commonly classified according to their mechanisms of action, with each drug class exerting distinct and often overlapping effects on ovarian structure and function. Although these agents differ in their primary anticancer mechanisms, many ultimately converge on common pathways of ovarian injury, including DNA damage, oxidative stress, mitochondrial dysfunction, and activation of programmed cell death [65].

##### 3.2.1.1. Alkylating Agents

Alkylating agents, including cyclophosphamide (CTX), busulfan, and platinum‐based compounds such as cisplatin and carboplatin, exert their cytotoxic effects by inducing deoxyribonucleic acid (DNA) alkylation and cross‐linking, ultimately leading to DNA strand breaks and cell death. Among all chemotherapeutic classes, alkylating agents are consistently associated with the highest risk of permanent ovarian insufficiency because of their direct toxicity toward dormant primordial follicles [67]. In addition to its antitumor efficacy, CTX is characterized by pronounced nonspecific toxicity, resulting in extensive depletion of primordial follicles and direct destruction of oocytes.

##### 3.2.1.2. Antimetabolites

Antimetabolites interfere with nucleotide synthesis and disrupt DNA and ribonucleic acid (RNA) integrity, thereby arresting cell division. Their ovarian toxicity is primarily directed toward metabolically active follicles, making growing follicular populations particularly susceptible [36].

##### 3.2.1.3. Antitumor Antibiotics

Antitumor antibiotics, such as dactinomycin and doxorubicin, inhibit Topoisomerase II activity, leading to cell cycle arrest and apoptosis. Their cytotoxicity is also mediated through excessive ROS generation and mitochondrial dysfunction, further amplifying follicular injury in both GCs and oocytes [66, 68].

##### 3.2.1.4. Tyrosine Kinase Inhibitors (TKIs) and Protein Kinase Inhibitors (PKIs)

TKIs and PKIs disrupt intracellular signaling pathways that regulate cellular growth and survival. Experimental evidence indicates that these agents impair follicular maturation and reduce overall ovarian function, particularly in animal models [36, 69, 70].

##### 3.2.1.5. Platinum‐Based Drugs

Platinum‐based agents, especially cisplatin, disrupt GC and oocyte homeostasis by inducing DNA damage and targeting critical intracellular organelles, including mitochondria and the endoplasmic reticulum [36]. In addition, platinum compounds are among the strongest inducers of DNA double‐strand breaks, thereby activating ATM‐ATR‐mediated DNA damage responses that promote apoptosis and follicular depletion [71]. Table 1 summarizes the main chemotherapeutic agents, their ovarian cellular targets, and the associated adverse effects on ovarian function.

**Table 1:** Chemotherapy‐induced ovarian toxicity.

Category	Drug/class	Primary ovarian targets	Predominant mechanism(s)	Functional outcome
Alkylating agents	Cyclophosphamide, busulfan, cisplatin, and carboplatin	Primordial follicles and oocytes	DNA alkylation and cross‐linking leading to DNA strand breaks, apoptotic cell death, and irreversible follicular depletion	Progressive loss of ovarian reserve and increased risk of premature ovarian insufficiency
Antimetabolites	Methotrexate, 5‐fluorouracil, and cytarabine	Growing follicles	Inhibition of DNA and RNA synthesis, resulting in cell‐cycle arrest and apoptosis of metabolically active follicular cells	Selective loss of growing follicles and compromised fertility
Antitumor antibiotics	Dactinomycin and doxorubicin	Granulosa cells and oocytes	Topoisomerase II inhibition and DNA damage, triggering apoptotic pathways in follicular cells	Follicular atresia and granulosa cell depletion
Signaling inhibitors	Tyrosine kinase inhibitors and protein kinase inhibitors	Follicular somatic cells	Disruption of intracellular signaling pathways governing cell survival, growth, and differentiation	Impaired follicular maturation and decline in ovarian functional capacity
Platinum‐based agents	Cisplatin and carboplatin	Granulosa cells, oocytes, mitochondria, and endoplasmic reticulum	DNA damage combined with organelle dysfunction, oxidative stress, and activation of apoptotic signaling	Extensive follicle loss and marked reduction in ovarian reserve
Shared molecular pathways	—	Granulosa cells and oocytes	Convergent activation of apoptosis, oxidative stress, ferroptosis, dysregulated autophagy, DNA damage responses, PTEN/PI3K/AKT/FOXO3a disruption, and inflammation	Accelerated follicular depletion and development of premature ovarian insufficiency

#### 3.2.2. Molecular Mechanisms of Ovarian Damage

**Figure 3:**
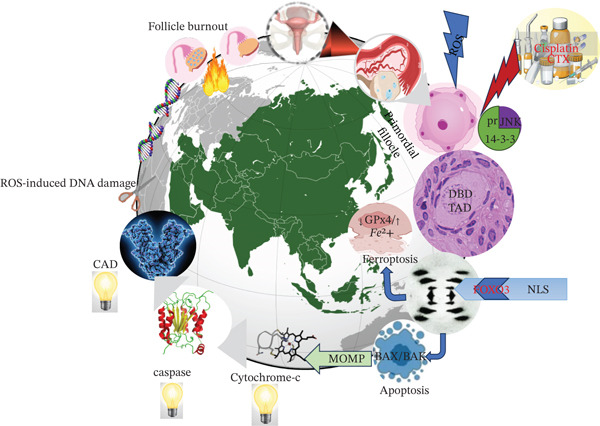
Integrated molecular mechanisms underlying chemotherapy‐induced ovarian injury. Chemotherapy initiates DNA damage, which subsequently triggers oxidative stress, mitochondrial dysfunction, apoptosis, ferroptosis, dysregulated autophagy, inflammatory signaling, and disruption of the PTEN/PI3K‐AKT‐FOXO3 pathway. The convergence of these interconnected mechanisms accelerates primordial follicle depletion and ultimately results in POI. Created by the author based on evidence synthesized from ([6, 34, 39, 55, 56, 58, 59, 71–80]).

As illustrated in Figure 3, chemotherapy‐induced ovarian injury results from a coordinated sequence of molecular events initiated by DNA damage and subsequently amplified through oxidative stress, mitochondrial dysfunction, apoptosis, ferroptosis, dysregulated autophagy, inflammatory signaling, and disruption of the PTEN/PI3K‐AKT‐FOXO3 pathway, ultimately leading to follicular depletion and POI [72].

##### 3.2.2.1. GC Apoptosis

Apoptosis represents one of the final common pathways through which chemotherapy‐induced molecular damage culminates in follicular loss. Rather than occurring as an isolated event, apoptotic signaling is initiated downstream of DNA damage responses, oxidative stress, mitochondrial dysfunction, and inflammatory activation, forming an integrated network that determines whether damaged follicles undergo repair or irreversible elimination [8, 72–74, 81]. Following chemotherapy exposure, DNA double‐strand breaks are rapidly sensed by ATM and ATR kinases, leading to activation of p53‐ and TAp63‐dependent checkpoint pathways. When genomic damage exceeds the repair capacity of oocytes and GCs, these signaling cascades initiate mitochondrial apoptosis [6, 71, 74, 75]. Chemotherapeutic agents such as CTX, cisplatin, and doxorubicin predominantly activate the intrinsic, mitochondria‐dependent apoptotic pathway by shifting the balance of Bcl‐2 family proteins toward proapoptotic members, including Bcl‐2‐associated X protein (Bax) and Bcl‐2 antagonist/killer (Bak). This imbalance increases mitochondrial membrane permeability, promotes the release of cytochrome c, and triggers activation of downstream effector caspases, particularly caspase‐9 and caspase‐3, ultimately resulting in GC death [34, 55, 56]. Consequently, apoptosis represents the downstream execution phase of DNA damage signaling rather than an independent pathogenic mechanism.

Under intense oxidative stress induced by chemotherapy, persistent ROS accumulation sustains JNK activation, establishing a feed‐forward loop that amplifies mitochondrial dysfunction and apoptotic signaling in GCs [58, 59]. This JNK activation then triggers a critical step that displaces FOXO1 from its cytoplasmic anchors, the 14‐3‐3 proteins. Once freed, FOXO1 translocates to the nucleus, where it can regulate the transcription of target genes [76]. In the nucleus, FOXO1 initiates a proapoptotic program. It upregulates key death‐promoting genes like Bim and Puma, which drive the loss of GCs and culminates in follicular atresia [58, 82].

In parallel, chemotherapy can also engage the extrinsic apoptotic pathway. Elevated ovarian expression of tumor necrosis factor‐*α* (TNF‐*α*) and Fas ligand (FasL) activates death receptor signaling in GCs, leading to caspase‐8 activation and further amplification of mitochondrial apoptotic signaling [83]. Crosstalk between extrinsic and intrinsic apoptotic pathways further amplifies follicular injury through mitochondrial membrane permeabilization and caspase cascade activation.

Importantly, GC loss is accompanied by reduced secretion of estrogen and AMH, creating a follicular microenvironment that is no longer supportive of oocyte survival. As a result, GC apoptosis not only disrupts follicular architecture but also indirectly accelerates oocyte loss and follicular atresia [64]. Loss of GC support also impairs oocyte metabolic homeostasis, increasing susceptibility to oxidative injury and DNA damage.

##### 3.2.2.2. Oxidative Stress and ROS Production

Oxidative stress acts as a major upstream driver of chemotherapy‐induced ovarian toxicity. Agents such as CTX and cisplatin disrupt redox homeostasis by markedly increasing the production of ROS within ovarian tissue [84–86]. As mitochondrial function deteriorates and endogenous antioxidant defenses become depleted, excessive ROS accumulates in GCs, leading to DNA damage, lipid peroxidation, loss of mitochondrial membrane potential, and activation of DNA damage response pathways. Oxidative stress serves as a central molecular hub linking DNA damage with multiple regulated cell‐death pathways. Excessive ROS not only activates mitochondrial apoptosis but also promotes lipid peroxidation, ferroptosis, inflammatory signaling, and impairment of autophagic homeostasis, thereby amplifying chemotherapy‐induced follicular injury [64].

Mitochondria are the cell′s main powerhouses, generating both energy and ROS. Under pathological conditions, such as exposure to chemotherapy, the mitochondrial electron transport chain (ETC) can become severely compromised. This disruption leads to increased electron leakage and an excessive production of ROS, contributing to cellular stress and damage [59, 87]. Moreover, damage to mitochondrial DNA (mtDNA) sets off a vicious cycle—as mitochondrial function declines, ROS production rises, which in turn drives further oxidative stress. This progressive damage accumulates over time, affecting both oocytes and surrounding GCs [59].

##### 3.2.2.3. Iron‐Dependent Ferroptosis in Chemotherapy‐Induced Ovarian Injury

In recent years, ferroptosis (an iron‐dependent and tightly regulated form of cell death) has emerged as an important contributor to chemotherapy‐induced ovarian injury. Accumulating evidence indicates that ferroptosis is not an isolated mechanism but develops downstream of chemotherapy‐induced oxidative stress and mitochondrial dysfunction, linking redox imbalance to irreversible follicular injury [77] Chemotherapeutic exposure disrupts intracellular iron homeostasis, leading to expansion of the labile iron pool (LIP) within GCs. Accumulation of Fe^2+^ facilitates Fenton chemistry, thereby promoting the generation of iron‐dependent ROS [88–92]. The resulting lipid peroxidation amplifies mitochondrial membrane damage and further increases intracellular ROS, thereby reinforcing apoptotic and inflammatory signaling.

Glutathione peroxidase 4 (GPX4) serves as the principal antioxidant enzyme protecting membrane phospholipids from iron‐dependent lipid peroxidation. Loss of GPX4 activity permits accumulation of phospholipid hydroperoxides, thereby initiating ferroptotic cell death [78]. Within the ovarian microenvironment, a primary enzymatic defense system (featuring superoxide dismutase (SOD) and catalase (CAT)) operates in a critical two‐step sequence. SOD first neutralizes superoxide anions (O2^-^), converting them to hydrogen peroxide (H₂O₂), which CAT then rapidly decomposes into harmless water and oxygen [93]. The exhaustion of these antioxidant defenses, specifically the loss of GPX4, strips follicles of their primary protection, leaving them acutely vulnerable to ferroptotic collapse in the wake of chemotherapy [78, 79].

ROS produced through lysosomal iron–mediated lipid peroxidation initiates widespread membrane damage, impairs mitochondrial function, and depletes cellular antioxidant defenses. Together, these events activate ferroptosis signaling pathways, accelerating GC death and destabilizing follicular homeostasis. Over time, this process contributes to the progressive depletion of the primordial follicle pool and ovarian reserve [94].

##### 3.2.2.4. Autophagy and Its Role in Chemotherapy‐Induced Ovarian Damage

Autophagy serves as a fundamental quality‐control process that supports follicular homeostasis under normal physiological conditions. Physiological autophagy is essential for maintaining mitochondrial integrity, preventing oxidative stress, and preserving oocyte quality. When tightly regulated, it enables cells to remove damaged components and maintain metabolic balance. Under chemotherapeutic stress, however, autophagy can become excessive or dysregulated, shifting from a protective mechanism to one that contributes to follicular loss. Persistent oxidative stress and DNA damage impair normal autophagic flux, converting autophagy from a cytoprotective process into a contributor to follicular degeneration. Consistent with this dual role, genetic deletion of essential autophagy‐related genes such as Atg7 has been shown to cause pronounced depletion of ovarian follicles and impaired fertility.

Chemotherapy further perturbs this process by inducing metabolic and oxidative stress and activating intracellular stress‐response pathways that disrupt normal autophagic flux. These observations indicate that dysregulated autophagy acts synergistically with apoptosis and ferroptosis to accelerate chemotherapy‐induced follicular depletion [34, 53].

##### 3.2.2.5. DNA Damage and Activation of Cell Death Pathways

Many chemotherapeutic agents, including cisplatin, CTX, and doxorubicin, directly induce DNA double‐strand breaks in both oocytes and GCs [73]. DNA double‐strand breaks represent one of the earliest molecular events following chemotherapy exposure and serve as the primary trigger initiating downstream stress‐response pathways. These lesions are detected by key DNA damage response kinases such as Ataxia Telangiectasia Mutated (ATM) and Ataxia Telangiectasia and Rad3‐related protein (ATR), which initiate downstream apoptotic and necroptotic signaling cascades [73]. Activation of ATM and ATR subsequently stimulates p53‐ and TAp63‐dependent checkpoint signaling, promoting either DNA repair or programmed cell death depending on the extent of genomic injury. Together, these molecular events establish the mechanistic link between genotoxic stress and the downstream activation of apoptosis, ferroptosis, and follicular burnout [73].

##### 3.2.2.6. The PTEN/PI3K/AKT/FOXO3a Axis and Primordial Follicle Activation

The PTEN/PI3K/AKT/FOXO3a axis serves as a central safeguard of primordial follicle quiescence and is a critical determinant of ovarian longevity. Under physiological conditions, phosphatase and tensin homolog (PTEN) restrains PI3K activity, thereby limiting AKT signaling and maintaining FOXO3a within the nucleus. In addition to regulating follicular quiescence, this pathway integrates signals derived from oxidative stress, DNA damage, and cellular metabolism, thereby coordinating adaptive responses that preserve ovarian homeostasis. In this state, FOXO3a drives transcriptional programs that suppress follicular activation and promote cellular survival [34, 95]. Consistent with this inhibitory role, pharmacological modulation of PI3K/AKT signaling has been shown to preserve the ovarian reserve. For instance, Urolithin A protects against chemotherapy‐induced follicle apoptosis by inhibiting PI3K/AKT signaling and preventing excessive primordial follicle activation [96]. Chemotherapy disrupts this tightly regulated balance by downregulating PTEN and activating AKT, leading to FOXO3a phosphorylation and nuclear exclusion. Concomitant activation of DNA damage signaling and oxidative stress further potentiates FOXO3 dysregulation, thereby amplifying primordial follicle activation.

Loss of nuclear FOXO3a removes inhibitory control over primordial follicles, triggering their synchronized activation, a phenomenon commonly referred to as primordial follicle burnout [97]. These follicles subsequently undergo uncontrolled growth followed by extensive atresia, resulting in rapid exhaustion of the ovarian reserve [98]. Rather than acting independently, PTEN/PI3K‐AKT‐FOXO3 dysregulation cooperates with DNA damage, oxidative stress, apoptosis, ferroptosis, and inflammatory signaling to accelerate depletion of the ovarian reserve [72].

##### 3.2.2.7. Inflammatory Response and Cytokine Signaling

Chemotherapy also profoundly alters the ovarian microenvironment by activating inflammatory signaling pathways. Inflammatory activation develops secondary to chemotherapy‐induced DNA damage, oxidative stress, and mitochondrial dysfunction, thereby reinforcing the network of molecular events responsible for ovarian injury rather than acting as an independent pathogenic process. Agents such as CTX and cisplatin increase intragonadal levels of proinflammatory cytokines, including Interleukin‐6 (IL‐6), Interleukin‐1 beta (IL‐1*β*), and TNF‐*α*. Beyond their direct cytotoxic effects on GCs, these cytokines drive the polarization of resident M0 macrophages toward a proinflammatory M1 phenotype, which serves as a major source of ROS and inflammatory mediators within the ovary [64, 84–86, 99]. Activated M1 macrophages further amplify oxidative stress through sustained ROS production, thereby promoting apoptosis, ferroptosis, and additional DNA damage within the follicular microenvironment.

The sustained release of ROS and cytokines from M1 macrophages creates a hostile follicular environment that promotes atresia of both primordial and growing follicles. When persistent, this inflammatory state (particularly in combination with oxidative stress) disrupts normal follicular dynamics, accelerates germ cell loss, and ultimately accelerates primordial follicle depletion, ovarian reserve exhaustion, and the development of POI [100].

Collectively, these findings demonstrate that chemotherapy‐induced ovarian toxicity is driven by a highly interconnected molecular network rather than by isolated pathogenic mechanisms. DNA damage represents the initiating event following chemotherapy exposure, triggering oxidative stress, mitochondrial dysfunction, apoptosis, ferroptosis, dysregulated autophagy, inflammatory signaling, and disruption of the PTEN/PI3K‐AKT‐FOXO3 pathway. The convergence of these processes accelerates primordial follicle depletion, compromises ovarian reserve, and ultimately leads to POI. Figure 3 summarizes these integrated molecular interactions, wheras Table 1 provides an overview of the major chemotherapeutic classes, their ovarian targets, and their predominant mechanisms of toxicity.

### 3.3. Chemotherapy‐Induced Morphological and Functional Alterations of the Ovary

Chemotherapeutic exposure leads to extensive structural and functional remodeling of ovarian tissue. These structural alterations represent the cumulative downstream consequence of the molecular events described in the previous section, including DNA damage, oxidative stress, apoptosis, ferroptosis, inflammatory remodeling, and dysregulation of the PTEN/PI3K‐AKT‐FOXO3 pathway. Evidence from both experimental models and clinical studies consistently shows a pronounced decline in primordial, growing, and Graafian follicles, reflecting substantial follicular depletion after treatment [34]. This reduction in follicle number is accompanied by notable architectural disturbances, including thinning of the GC layer and a decrease in corpus luteum volume, indicative of disrupted follicular maturation and impaired luteal function [34].

Functionally, these morphological changes manifest as a reduction in ovarian weight and a marked disturbance of endocrine homeostasis, characterized by decreased circulating levels of E2 and AMH, together with compensatory increases in FSH and LH [34]. In parallel, chemotherapy promotes excessive extracellular matrix deposition and progressive ovarian fibrosis, a process largely driven by activation of the transforming growth factor‐*β*1 (TGF‐*β*1) signaling pathway, which further compromises ovarian plasticity and overall functional integrity [101, 102]. Persistent ovarian fibrosis may further impair follicular regeneration by disrupting stromal architecture and ovarian vascularization, thereby aggravating long‐term reproductive dysfunction [102, 103].

### 3.4. POI

Chemotherapy‐induced ovarian damage arises from a complex interaction between genetic susceptibility and treatment‐related factors, including patient age [104], cumulative drug dose, combination chemotherapy regimens, treatment duration, and the extent of radiotherapy exposure [34]. The relative contribution of each factor varies substantially among individuals, suggesting that inherited genetic susceptibility may modify the ovarian response to chemotherapy. Together, these variables promote follicular atrophy and cellular injury, ultimately disrupting sex hormone secretion and giving rise to a spectrum of reproductive consequences, such as infertility, amenorrhea, and other clinical manifestations of ovarian insufficiency [11, 12, 15–17, 36, 64, 94, 105–107].

The clinical expression of ovarian injury is highly variable. It may manifest as diminished ovarian reserve, chemotherapy‐induced amenorrhea (CIA) [27], or POI, which is defined as a decline in ovarian function occurring before the expected age of natural menopause. In its most severe form, ovarian damage may progress to POF, characterized by profound ovarian dysfunction, hypoestrogenism, elevated gonadotropin levels (FSH > 40 IU/L) [50], as well as irreversible loss of fertility before or by the age of 40 [34, 108]. Importantly, not all women exposed to comparable chemotherapy regimens develop POI, indicating that biological susceptibility likely modifies treatment‐related ovarian toxicity.

### 3.5. The Central Role of the PI3K/AKT/FOXO3 Axis in Asian Populations

The FOX is a key downstream transcription factor of the PI3K–AKT signaling pathway and serves as a master regulator of primordial follicle quiescence, cellular stress responses, DNA repair, and ovarian longevity [39].

Although multiple modifications regulate FOXO3, its fate under chemotherapy hinges on phosphorylation. This specific modification controls FOXO3′s movement into the nucleus, where it then directs the transcription of target genes. Phosphorylation within the nucleus promotes FOXO3 export to the cytoplasm, thereby suppressing its transcriptional activity, whereas cytoplasmic phosphorylation facilitates its nuclear localization and functional activation. Through this dynamic regulation, FOXO3 governs a broad spectrum of cellular processes, including inflammation, aerobic glycolysis, autophagy, apoptosis, oxidative stress responses, cell‐cycle arrest, and DNA repair [39]. These regulatory mechanisms are particularly relevant during chemotherapy, where DNA damage and oxidative stress dynamically alter FOXO3 activity, thereby influencing follicular survival or depletion.

Beyond its role in cellular homeostasis, FOXO3 has been implicated in the modulation of diverse disease processes. It contributes to delayed telomere attrition, enhanced cellular self‐renewal, and preservation of genomic stability, collectively conferring protection against aging‐related decline. These observations underscore the critical importance of FOXO3 expression levels and post‐translational modifications in maintaining tissue integrity and mitigating age‐associated pathologies [39].

At the genomic level, the human FOXO3 gene is located on Chromosome 6 (6q21) and consists of four exons separated by three introns [39, 109]. The FOXO3 protein contains five conserved functional domains, including a DNA‐binding domain (DBD), two nuclear localization signals (NLSs), a nuclear export signal (NES), and a C‐terminal transactivation domain (TAD) [80]. These domains collectively regulate FOXO3 transcriptional activity and intracellular trafficking. Notably, interaction with the chaperone protein 14‐3‐3 enhances FOXO3 nuclear export and prevents its re‐entry into the nucleus, serving as a key regulatory checkpoint of FOXO3 signaling [39, 80].

Collectively, these functions highlight the central role of FOXO3 in maintaining cellular resilience during aging and stress. FOXO3 activity is tightly regulated through nucleocytoplasmic shuttling, a process controlled by AKT‐mediated phosphorylation and interaction with 14‐3‐3 proteins.

A substantial body of evidence has established strong associations between single‐nucleotide polymorphisms (SNPs) within the insulin/IGF‐1 signaling pathway and human longevity [110]. Central to this pathway is FOXO3a, an evolutionarily conserved transcription factor that integrates PI3K/AKT signaling to regulate cellular metabolism, oxidative stress responses, apoptosis, and senescence [111–113]. Meta‐analyses across diverse populations have identified the G allele of SNP rs2802292 as a significant determinant of exceptional longevity (OR = 1.12), functioning as a cisexpression quantitative trait locus (cis‐eQTL) that modulates FOXO3‐dependent regulatory networks [114, 115]. Among the several FOXO3 variants reported in longevity genome‐wide association study (GWAS), rs2802292 was prioritized because it has the strongest replicated evidence and the most consistent effect in Asian populations.

This allele is associated with enhanced stress resistance and reduced cardiovascular risk, with its protective effects being most pronounced in individuals aged 95 years and older [116]. Importantly, large‐scale GWASs conducted in Han Chinese and Japanese centenarian cohorts have consistently reinforced the role of FOXO3a variants in longevity among Asian populations, highlighting their contribution to organismal homeostasis and resistance to age‐related diseases [111]. Genetic‐marker‐based prediction of chemotherapy‐induced ovarian toxicity has already been explored using variants such as CYP3A4 ^∗^1B and GSTA1 as a complement to conventional ovarian‐reserve testing (AMH, AFC) [117]. This precedent suggests that genotyping for the rs2802292 FOXO3 longevity allele described above could similarly be investigated as a testable, population‐specific biomarker for pretreatment risk stratification in Asian women receiving gonadotoxic chemotherapy, pending prospective validation [117].

Beyond FOXO3, AKT1 emerges as a critical downstream effector within this signaling axis, exhibiting pronounced ethnic variability in its genetic architecture. In Chinese populations, SNPs such as rs2498786, rs2494752, and rs5811155 have been associated with susceptibility to microscopic polyangiitis (MPA), with specific alleles (rs2498786 G, rs2494752 G, and rs5811155 insT) conferring protective effects [118].

Notably, the frequencies of these protective AKT1 variants are substantially lower in East Asian populations than in European cohorts (1000 genomes/gnomAD), paralleling the higher prevalence of MPA observed in Chinese and Japanese individuals [118–120]. Further evidence of population‐specific divergence is provided by the locus 5q33.3 (rs2149954), which is strongly associated with survival beyond 90 years in Han Chinese populations (OR = 1.10, *p* = 1.74 × 10^−8^), yet exhibits marked differences in allele frequency and functional relevance between Japanese (24%) and European (43%) populations [111, 121]. Such disparities in linkage disequilibrium patterns and allele distribution indicate that longevity‐associated SNPs identified in one ethnic group may not exert equivalent biological effects in others, including African American or Hispanic populations [116].

These findings suggest that the PI3K/AKT/FOXO3 signaling axis in Han Chinese and Japanese populations harbors distinct genetic variants that shape interindividual differences in cellular resilience to stress and damage [34, 111, 122]. The unique distribution of these variants, together with their interaction with the anti‐aging KLOTHO pathway, implies that the ovarian and systemic regulatory environment in Asian populations may operate within a narrower and more sensitive threshold [111].

Although FOXO3 polymorphisms have been consistently associated with exceptional longevity in Asian populations, direct evidence linking these variants to chemotherapy‐induced ovarian toxicity remains limited. However, no clinical studies have yet directly demonstrated that these variants alter the risk of chemotherapy‐induced POI, emphasizing the need for prospective genotype‐stratified investigations. Nevertheless, because FOXO3 regulates DNA repair, oxidative stress responses, apoptosis, and primordial follicle activation, it is biologically plausible that genetic variants altering FOXO3 activity may also modify ovarian susceptibility to chemotherapy‐induced injury. This mechanistic rationale forms the conceptual basis of the present two‐hit hypothesis rather than representing an established clinical conclusion.

Under oxidative stress, FOXO transcription factors are not only translocated into the nucleus but also upregulated via a JNK‐dependent self‐regulatory loop [123]. FOXO1, in particular, can bind directly to its own promoter to amplify its expression under stress [58]. This positive feedback may be especially relevant in Asian populations, where genetic variations may predispose individuals to a more rapid activation of this pathway, thereby hastening ovarian reserve depletion [58].

**Figure 4:**
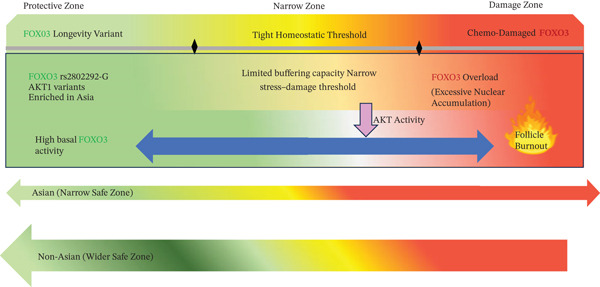
A two‐hit model of population‐specific susceptibility to chemotherapy‐induced premature ovarian insufficiency. Created by the author based on evidence synthesized from ([11, 98, 111–116, 118–122]).

Accordingly, as illustrated in Figure 4, this heightened genetic susceptibility may render the PI3K/AKT/FOXO3 axis particularly vulnerable to exogenous stressors such as chemotherapy, thereby accelerating follicular depletion and increasing the risk of POI. The currently available molecular, genetic, and population‐based evidence supports a biologically plausible (but not yet clinically proven) framework in which inherited susceptibility and chemotherapy‐induced cellular stress cooperate to accelerate ovarian reserve depletion.

Although the genetic evidence that has been presented is compelling, we must acknowledge that a lack of longitudinal clinical data across many Asian regions remains a challenge. This gap is largely a reflection of the reality that high‐quality cancer registries and robust genomic databases are still emerging in many low and middle‐income countries. As a result, the true scale of ethnic‐specific vulnerability to chemotherapy‐induced ovarian damage may be significantly underreported [11].

**Figure 5:**
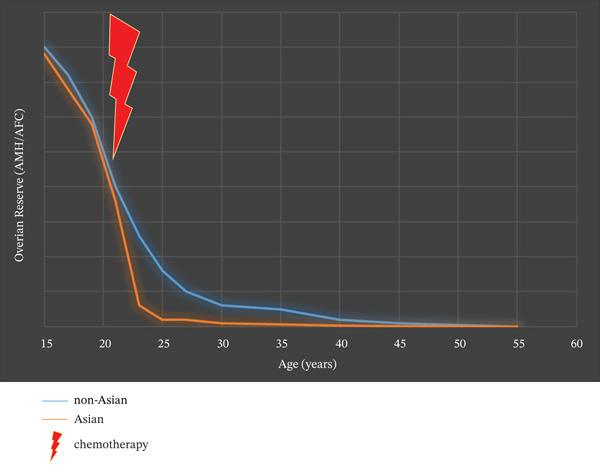
A comparative schematic of accelerated ovarian reserve decline in Asian and non‐Asian populations following chemotherapy. Created by the author based on evidence synthesized from ([4–7, 9, 11, 29, 33, 36, 39, 111]).

This highlights a critical need for more localized data collection and broader global collaboration to ensure that precision medicine is truly inclusive. As illustrated in Figure 5, which presents a comparative schematic of accelerated ovarian reserve depletion following chemotherapy in Asian versus non‐Asian populations, future multicenter longitudinal studies integrating genomic profiling, functional validation, and prospective reproductive outcomes across diverse Asian populations will be essential to determine whether the proposed two‐hit model can be translated into genotype‐informed fertility preservation and personalized oncologic care.

## 4. Conclusion

This review integrates current molecular, experimental, and population‐based evidence to examine the mechanisms underlying chemotherapy‐induced ovarian injury and the potential contribution of the PI3K/AKT/FOXO3 signaling axis to population‐specific ovarian susceptibility. It first outlines normal ovarian physiology and highlights the central role of the PI3K/AKT/FOXO3 signaling pathway in maintaining cellular homeostasis, regulating follicular development, and preserving the ovarian reserve. Mechanistic evidence consistently demonstrates that this pathway is integral to ovarian integrity through its coordinated regulation of autophagy, apoptosis, oxidative stress, and cellular senescence, while maintaining primordial follicle quiescence, coordinating stress responses, and preserving long‐term ovarian function.

Accumulating data further indicate that chemotherapeutic agents profoundly disrupt the PI3K/AKT/FOXO3 axis, activating multiple deleterious pathways, including apoptosis, ferroptosis, DNA damage responses, and inflammatory signaling. These disruptions contribute to progressive follicular depletion and increasing risk of POI. Given the rising cancer burden and aging demographics across Asian populations, the preservation of fertility following cancer treatment has emerged as a critical clinical priority.

Genetic studies conducted in several Asian populations have identified population‐specific variants within the PI3K/AKT/FOXO3 pathway that have been associated with longevity and cellular stress responses. Because these pathways also regulate DNA repair, oxidative stress, apoptosis, and primordial follicle activation, they provide a biologically plausible basis for investigating whether inherited genetic variation influences susceptibility to chemotherapy‐induced ovarian injury. Moreover, interactions between this axis, longevity‐associated mechanisms, and the KLOTHO antiaging pathway suggest that both ovarian and systemic regulatory environments in Asian populations may operate within a narrower homeostatic threshold.

The available evidence supports a conceptual framework in which inherited genetic susceptibility and chemotherapy‐induced cellular stress may interact to accelerate ovarian reserve depletion in susceptible individuals. Accordingly, the proposed two‐hit hypothesis should be regarded as a testable mechanistic model rather than an established clinical conclusion. Future experimental studies, prospective cohort investigations, and genotype‐stratified clinical trials will be required to determine whether this hypothesis can be translated into clinical practice.

If validated, this framework could support genotype‐informed risk stratification, individualized fertility‐preservation strategies, and optimization of chemotherapy protocols for women at increased genetic risk, particularly within Asian populations. Such genotype‐informed data could be integrated into the pretreatment fertility‐counseling and risk‐stratification pathway recently updated by the American Society of Clinical Oncology (ASCO). However, because direct clinical evidence linking these variants to POI risk remains limited, such screening should currently be considered exploratory rather than standard practice, and prospective studies are needed to validate whether these genetic markers can inform individualized fertility‐preservation decisions.

Ultimately, integrating molecular genetics with reproductive oncology may provide the foundation for precision fertility preservation, enabling future interventions that protect ovarian function while maintaining effective anticancer therapy across genetically diverse populations. Consistent with the narrative, nonsystematic nature of this review, formal risk‐of‐bias tools were not applicable, and quality assessment was instead guided by recognized standards for narrative reviews (SANRA).

**Nomenclature**
AFC: antral follicle count
AMH: anti‐Müllerian hormone
AKT: protein kinase B
ATM: Ataxia Telangiectasia Mutated
ATR: ataxia telangiectasia and rad3‐related protein
BAK: Bcl‐2 antagonist/killer
Bax: Bcl‐2‐associated X protein
Bcl‐2: B‐cell lymphoma 2
CAT: catalase
CIA: chemotherapy‐induced amenorrhea
cis‐eQTL: cisexpression quantitative trait locus
CTX: cyclophosphamide
DBD: DNA‐binding domain
DNA: deoxyribonucleic acid
E2: estradiol
ECM: extracellular matrix
eQTL: expression quantitative trait locus
ETC: electron transport chain
FasL: Fas ligand
FOXO3/FOXO3a: forkhead box O3 transcription factor
FSH: follicle‐stimulating hormone
GC: granulosa cell
GPx4: glutathione peroxidase 4
GWAS: genome‐wide association study
IGF‐1: insulin‐like growth factor 1
IL‐1*β*
: interleukin‐1 beta
IL‐6: interleukin‐6
JNK: c‐Jun N‐terminal kinase
LH: luteinizing hormone
LIP: labile iron pool
MOMP: mitochondrial outer membrane permeabilization
MPA: microscopic polyangiitis
mtDNA: mitochondrial DNA
NES: nuclear export signal
NLS: nuclear localization signal
PI3K: phosphoinositide 3‐kinase
PKIs: protein kinase inhibitors
POF: premature ovarian failure
POI: premature ovarian insufficiency
PTEN: phosphatase and tensin homolog
RNA: ribonucleic acid
ROS: reactive oxygen species
SIRT1: sirtuin 1
SNPs: single nucleotide polymorphisms
SOD: superoxide dismutase
TAD: transactivation domain
TGF‐*β*1: transforming growth factor‐beta 1
TKIs: tyrosine kinase inhibitors
TNF‐*α*
: tumor necrosis factor‐alpha

## Author Contributions

The author solely conceived and designed the study, conducted the literature review and data curation, developed the computational framework, performed the analysis, interpreted the results, and wrote and revised the manuscript.

## Funding

No funding was received for this manuscript.

## Disclosure

The author has read and approved the final version of the manuscript.

## Ethics Statement

This study was conducted as a computational, mechanistic integrative simulation based exclusively on previously published experimental, clinical, and molecular data. No new human participants, identifiable personal data, or animal subjects were involved in the conduct of this research. All data sources were obtained from publicly accessible, peer‐reviewed literature, and were used in accordance with applicable guidelines and standards for secondary data analysis. As such, formal ethical approval and informed consent to participate were not required. The study was carried out with careful consideration of research integrity principles, ensuring appropriate citation, responsible data handling, and respect for the original sources of all incorporated findings.

## Consent

This manuscript does not contain any individual or person′s data in any form, including identifiable images, videos, or personal information. All information presented in this study is derived from previously published, anonymized, and publicly available sources. Therefore, consent for publication is not applicable.

## Conflicts of Interest

The author declares no conflicts of interest.

## Supporting information


**Supporting Information** Additional supporting information can be found online in the Supporting Information section. Table S1: (PRISMA). An adapted PRISMA 2020 checklist documenting the literature identification, screening, and synthesis process followed in this structured narrative review, together with an explicit indication of checklist items (e.g., risk‐of‐bias assessment, quantitative synthesis, certainty assessment) not applicable to a nonsystematic, hypothesis‐generating review design.

## Data Availability

All data generated or analyzed during this study are included in this published article.
