# Supplementary material for: A Two‐Hit Hypothesis for Chemotherapy‐Induced Primary Ovarian Insufficiency in Asian Populations: A Population‐Specific Mechanistic Framework Linking Genetic Susceptibility and Cytotoxic Stress via the PI3K‐AKT‐FOXO3 Axis
Source: Biomed Res Int. 2026 Jul 25;2026:2340719. doi: 10.1155/bmri/2340719 (PMC13401246; doi:10.1155/bmri/2340719)
Supplement: Supplementary file 1 — Supporting Information Additional supporting information can be found online in the Supporting Information section. Table S1: (PRISMA). An adapted PRISMA 2020 checklist documenting the literature identification, screening, and synthesis process followed in this structured narrative review, together with an explicit indication of checklist items (e.g., risk‐of‐bias assessment, quantitative synthesis, certainty assessment) not applicable to a nonsystematic, hypothesis‐generating review design. [file BMRI-2026-2340719-s001.docx]

**PRISMA 2020 Checklist (Expanded, Adapted for a Structured Narrative Review)**

*Manuscript: A Two-Hit Hypothesis for Chemotherapy-Induced POI in Asian Populations: A Population-Specific Mechanistic Framework Linking Genetic Susceptibility and Cytotoxic Stress via the PI3K-AKT-FOXO3 Axis*

This manuscript is a structured narrative review synthesizing human clinical, population-based, animal, and mechanistic evidence on chemotherapy-induced ovarian toxicity and premature ovarian insufficiency (POI), proposed as a two-hit hypothesis integrating longevity-associated genetic susceptibility (PI3K-AKT-FOXO3 axis) with chemotherapy-induced cytotoxic stress in Asian populations. It is not a systematic review or meta-analysis and was not prospectively registered. The PRISMA 2020 checklist is adapted here to maximize methodological transparency for the literature identification, screening, and synthesis process, while explicitly noting the checklist items (e.g., risk-of-bias assessment, quantitative synthesis, certainty assessment) that are not applicable to a narrative-review design.

| **Item #** | **Section / Topic** | **Checklist Item** | **Location in Manuscript** |
| --- | --- | --- | --- |
| **TITLE** | | | |
| **1** | **Title** | - Identify the report as a narrative review examining a proposed mechanistic hypothesis. - Title states population (Asian populations), exposure (chemotherapy), outcome (POI), and the mechanistic pathway (PI3K-AKT-FOXO3 axis). | *Title page: “A Two-Hit Hypothesis for Chemotherapy-Induced POI in Asian Populations: A Population-Specific Mechanistic Framework Linking Genetic Susceptibility and Cytotoxic Stress via the PI3K-AKT-FOXO3 Axis.”* |
| **ABSTRACT** | | | |
| **2** | **Abstract** | - Background, rationale, methodological approach, key mechanistic findings, and conclusions reported in structured form. - Hypothesis-generating nature of the two-hit model explicitly stated; direct clinical validation noted as limited. - All abbreviations spelled out at first use. | *Abstract, paragraphs 1-3.* |
| **INTRODUCTION** | | | |
| **3** | **Rationale** | Describes the rising burden of cancer and chemotherapy-induced ovarian toxicity in Asian populations, the regulatory role of the PI3K-AKT-FOXO3 axis, and the knowledge gap regarding population-specific genetic susceptibility. | *Introduction, paragraphs 1-4.* |
| **4** | **Objectives** | States the aim of synthesizing mechanistic, experimental, and population-based evidence to propose and support a two-hit hypothesis linking longevity-associated genetic variants and chemotherapy-induced cellular stress. | *Introduction, final paragraph; Abstract.* |
| **METHODS** | | | |
| **5** | **Eligibility criteria** | - Eligible: peer-reviewed, English-language human clinical/population studies, animal/experimental models, and mechanistic studies directly relevant to chemotherapy-induced ovarian injury, POI, or the PI3K/AKT/FOXO3 axis. - Excluded: duplicate records, conference abstracts without full text, editorials, commentaries, non-English publications, and studies without direct mechanistic or clinical relevance. | *Methodology, paragraphs 4-5.* |
| **6** | **Information sources** | Records retrieved from PubMed, Web of Science, and Google Scholar, supplemented by reference-list/citation searching; records published from 2004 onward, with emphasis on 2015- 2025 literature. | *Methodology, paragraph 2.* |
| **7** | **Search strategy** | Full Boolean search strings reported, combining core terms (FOXO3, AKT, PI3K, chemotherapy, ovary, POI, oxidative stress, aging/longevity, cancer, Asian populations) with MeSH terms where appropriate; representative queries listed in full. | *Methodology, paragraph 3 (bulleted search strings).* |
| **8** | **Selection process** | Records screened against predefined eligibility criteria; duplicates, non-English records, conference abstracts, and mechanistically irrelevant records excluded. Single-author screening is disclosed as a design limitation. | *Methodology, paragraph 6; Limitations statement, Discussion/Conclusion.* |
| **9** | **Data collection process** | Data items extracted narratively from each eligible study by the author; no automated or dual-reviewer extraction tool was used, consistent with the single-author narrative-review design. | *Methodology, paragraph 6.* |
| **10a/10b** | **Data items** | - Data extracted: study design, experimental model or study population, chemotherapeutic agent(s) evaluated, molecular pathway(s) investigated, reproductive/ovarian outcomes, and principal mechanistic findings. - No outcome-effect estimates were extracted for pooling, consistent with the narrative (non-meta-analytic) synthesis design. | *Methodology, paragraph 6; Table 1 (chemotherapeutic agents, cellular targets, and adverse ovarian effects).* |
| **11** | **Study risk-of-bias assessment** | Not applicable. Formal risk-of-bias/quality-appraisal tools (e.g., Cochrane RoB2, ROBINS-I, SYRCLE) were not applied, as this manuscript is designed and presented as a structured narrative review rather than a systematic review or meta-analysis. This scope limitation is explicitly acknowledged in the Methodology. | *Methodology, paragraph 7 (design/scope statement).* |
| **12** | **Effect measures** | Not applicable, no pooled effect measures were calculated; findings are synthesized narratively rather than quantitatively. | *Not applicable.* |
| **13a–13f** | **Synthesis methods** | Not applicable, no statistical synthesis, meta-analysis, or quantitative pooling was performed. Evidence was synthesized narratively based on consistency of findings across experimental, clinical, and population-based studies. | *Methodology, final paragraph.* |
| **14** | **Reporting bias assessment** | Not applicable, no meta-analytic synthesis was performed; publication-bias statistics (e.g., funnel plots, Egger's test) do not apply to a narrative synthesis. | *Not applicable.* |
| **15** | **Certainty assessment** | Not applicable, formal certainty-of-evidence frameworks (e.g., GRADE) are designed for pooled quantitative outcomes and were not applied to this narrative, hypothesis-generating synthesis. | *Not applicable.* |
| **16a** | **Study selection** | PRISMA 2020 flow diagram reports: records identified (n=230: 195 via database searching, 35 via reference-list/citation searching), duplicates removed (n=36), records screened (n=194), records excluded at screening (n=47), full-text articles assessed (n=147), full-text exclusions with reasons (n=24), and studies included in the narrative synthesis (n=123). | *Methodology, Figure 1 and accompanying text; supplementary PRISMA 2020 flow diagram.* |
| **16b** | **Study selection – exclusions** | - Full-text exclusion reasons reported**:** not related to ovarian toxicity (n=6); not related to PI3K/AKT/FOXO3 signaling (n=5); conference abstract/no full text (n=5); no mechanistic or clinical relevance (n=4); non-English language (n=4). | *Supplementary PRISMA 2020 flow diagram.* |
| **17** | **Study characteristics** | Included studies span human clinical/population studies, animal and experimental models, and in vitro mechanistic studies; the majority focus on East and South Asian cohorts (China, Japan, South Korea, India, Southeast Asia), with comparative non-Asian (European, African) evidence also reviewed. | *Methodology, paragraphs 5-6; Discussion, throughout.* |
| **18** | **Risk of bias in studies** | Not applicable, consistent with Item 11, no formal risk-of-bias assessment was performed. | *Not applicable.* |
| **19** | **Results of individual studies** | Individual study findings are synthesized narratively within thematic subsections (ovarian physiology, chemotherapy-induced molecular injury, DNA-damage response, apoptosis, oxidative stress, ferroptosis, and population-specific genetic susceptibility) rather than tabulated by individual study effect estimates. | *Discussion, all subsections; Table 1.* |
| **20a–20d** | **Results of syntheses** | Not applicable in the meta-analytic sense, no statistical pooling was performed. Narrative convergence of evidence across study types is presented instead, with consistency and gaps in the evidence explicitly discussed. | *Discussion, throughout; Conclusion.* |
| **21** | **Reporting biases** | Not applicable, no quantitative synthesis was performed for which reporting bias could be statistically assessed. | *Not applicable.* |
| **22** | **Certainty of evidence** | The strength/certainty of evidence for each major claim is conveyed narratively (e.g., explicit statements that direct clinical validation of FOXO3/AKT variants in chemotherapy-induced POI is currently lacking, and that the two-hit model is hypothesis-generating rather than clinically established). | *Abstract, paragraph 3; Discussion (FOXO3 longevity-variant subsection); Conclusion, paragraphs 3-4.* |
| **DISCUSSION** | | | |
| **23a** | **Discussion – interpretation** | Results interpreted in the context of established ovarian-aging and follicular-quiescence frameworks; mechanistic pathways (DNA damage, oxidative stress, apoptosis, ferroptosis, autophagy, inflammation) are explicitly linked as a coordinated, sequential injury cascade converging on the PI3K-AKT-FOXO3 axis. | *Discussion, “Molecular Mechanisms of Ovarian Damage.”* |
| **23b** | **Discussion – limitations of evidence** | Explicitly discusses the lack of direct clinical/experimental validation linking FOXO3/AKT variants to chemotherapy-induced POI, the paucity of longitudinal data from several Asian regions, and the absence of formal risk-of-bias assessment given the narrative-review design. | *Discussion, FOXO3 longevity-variant subsection; Conclusion, paragraph 4.* |
| **23c** | **Discussion – limitations of process** | Single-author literature screening, non-registration of the review protocol, and the narrative (non-systematic, non-meta-analytic) design are disclosed as methodological limitations. | *Methodology, paragraph 7; Conclusion, paragraph 4.* |
| **23d** | **Discussion – implications** | Implications framed as conditional/future-oriented: if validated, the two-hit framework could support genotype-informed risk stratification, individualized fertility-preservation counselling, and comparative evaluation of chemotherapy regimens in genetically susceptible individuals; prospective genotype-stratified studies are called for. | *Conclusion, final three paragraphs.* |
| **OTHER INFORMATION** | | | |
| **24a** | **Registration** | This narrative review was not prospectively registered (e.g., PROSPERO), as it was conceived as a hypothesis-generating mechanistic synthesis rather than a systematic review intended for evidence-based guideline development. | *Methodology, paragraph 7 (design/scope statement).* |
| **24b** | **Protocol** | No formal review protocol was prepared prior to conducting the review. The complete search, eligibility, and synthesis methodology is described in full in the Methodology section. | *Methodology, entire section.* |
| **24c** | **Amendments** | Not applicable, no protocol was registered; no amendments to report. | *Not applicable.* |
| **25** | **Support** | No external funding was received for this study. | *Declarations section.* |
| **26** | **Competing interests** | The author declares no competing interests, financial or non-financial. | *Declarations section.* |
| **27** | **Availability of data / code** | No new primary datasets or code were generated; all data supporting the narrative synthesis are the published sources cited in the reference list (123 unique references). The completed PRISMA 2020 checklist and flow diagram are provided as supplementary files. | *Declarations section; supplementary files.* |

**PRISMA 2020 Flow Diagram**

*The counts below reconstruct the study-selection pathway consistent with the 230 potentially relevant records described in the Methodology. These figures should be verified and, if necessary, adjusted by the author against her original search and screening records before final submission, as the original review did not track screening numbers at each stage in the initial manuscript version.*


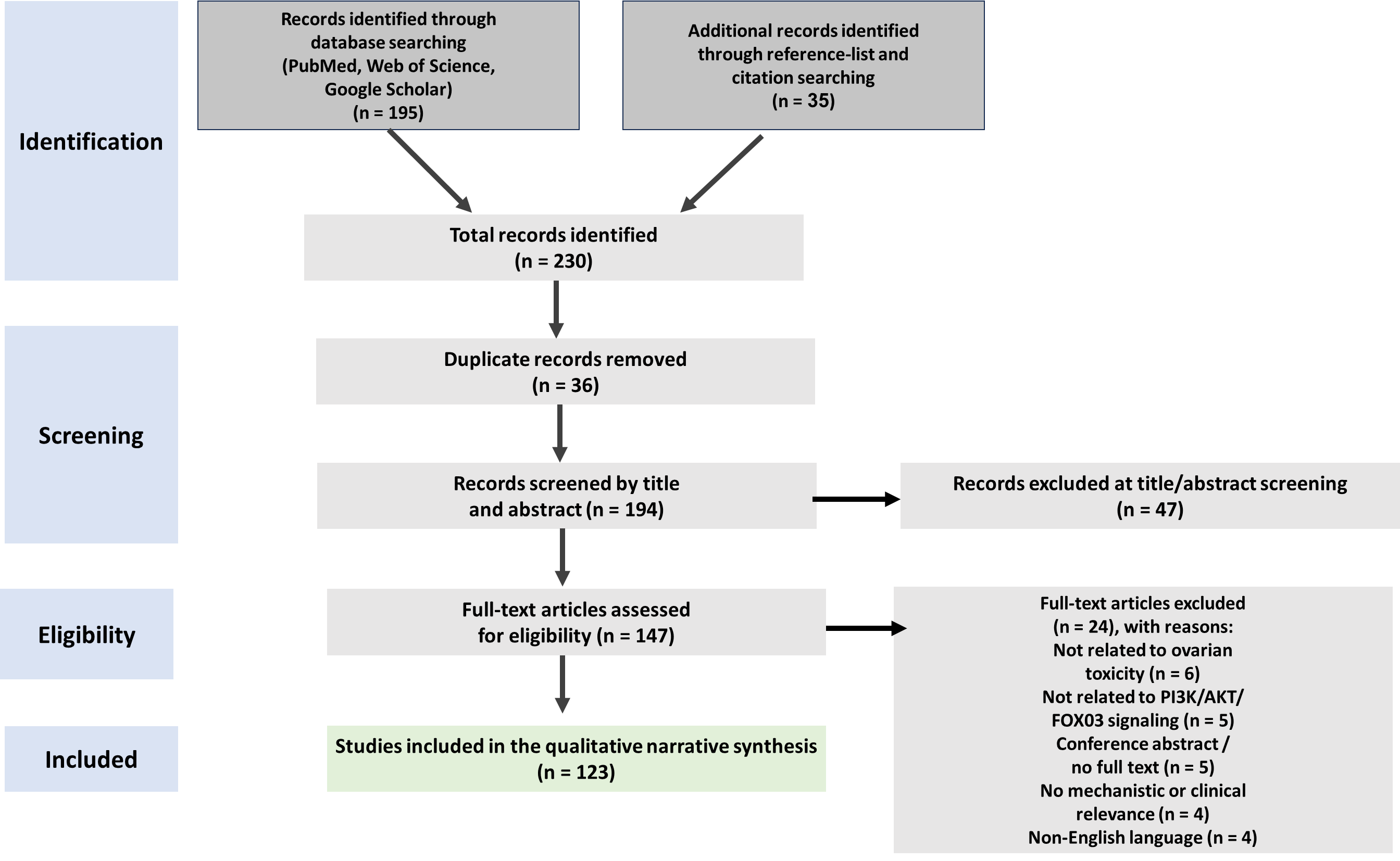


*Reference: Page MJ, McKenzie JE, Bossuyt PM, et al. The PRISMA 2020 statement: an updated guideline for reporting systematic reviews. BMJ 2021;372:n71. doi:10.1136/bmj.n71*
